# *POU1F1* is a novel fusion partner of *NUP98* in acute myeloid leukemia with t(3;11)(p11;p15)

**DOI:** 10.1186/1476-4598-12-5

**Published:** 2013-01-18

**Authors:** Susana Lisboa, Nuno Cerveira, Susana Bizarro, Cecília Correia, Joana Vieira, Lurdes Torres, José M Mariz, Manuel R Teixeira

**Affiliations:** 1 Department of Genetics, Portuguese Oncology Institute, Rua Dr. António Bernardino de Almeida, 4200-072, Porto, Portugal; 2 Department of Onco-Hematology, Portuguese Oncology Institute, Porto, Portugal; 3 Institute of Biomedical Sciences (ICBAS), University of Porto, Porto, Portugal

**Keywords:** *NUP98 gene*, *NP98-POU1F1*, Gene fusion, Acute leukemia

## Abstract

**Background:**

*NUP98* gene rearrangements have been reported in acute myeloid leukemia, giving rise to fusion proteins that seem to function as aberrant transcription factors, and are thought to be associated with poor prognosis.

**Findings:**

A patient with treatment-related acute myeloid leukemia presented a t(3;11)(p11;p15) as the only cytogenetic abnormality. FISH and molecular genetic analyses identified a class 1 homeobox gene, *POU1F1*, located on chromosome 3p11, as the fusion partner of *NUP98*. In addition, we have found that the patient harbored an *FLT3-ITD* mutation, which most likely collaborated with the *NUP98-POU1F1* fusion gene in malignant transformation.

**Conclusions:**

We have identified *POU1F1* as the *NUP98* fusion partner in therapy-related AML with a t(3;11)(p11;p15). This is the first POU family member identified as a fusion partner in human cancer.

## Findings

Acute myeloid leukemia (AML) is often associated with chromosomal translocations, resulting in fusion genes that have implications in disease prognosis and treatment. Chromosomal translocations involving the *NUP98* gene have been reported in a wide range of hematopoietic malignancies, involving more than 20 different partner genes to generate fusion proteins with abnormal function [1]. The frequency of these rearrangements in AML is 1 to 2% and they seem to be associated with poor prognosis, thus highlighting the relevance of identifying and characterizing cases harboring such genetic alterations [1,2].

The *NUP98* gene codes for a protein that is a component of the nuclear pore complex (NPC) and contains multiple nontandem GLFG repeats (Gly-Leu-Phe-Gly) that are thought to function as docking sites to allow the bidirectional transport of mRNA and proteins between the nucleus and the cytoplasm [1]. The NUP98 protein is also involved in cell cycle progression, mitotic spindle formation, and gene transcription [1]. All the translocations so far described involving the *NUP98* gene result in the fusion of its 5^′^ region (coding for the GLFG repeats) to the 3^′^ region of the partner gene. The C-terminal partners can be divided into two general classes: homeodomain (HD) proteins and non-HD proteins [1]. HD proteins contain a DNA-binding domain (the HD domain) that is in all instances retained in the fusion protein, with its amino terminal region replaced by the GLFG repeats of NUP98. The fusion protein seems to function as an aberrant transcription factor, directly binding DNA to activate gene transcription and leading to the deregulation of *HOXA* cluster genes that are important for normal hematopoietic differentiation [1,3].

A 57-year-old female was diagnosed with a breast adenocarcinoma in 2001 (T1N0M0; treated with radical mastectomy, followed by four courses of chemotherapy with 5-fluorouracil, epirubicin, and cyclophosphamide, radiotherapy and hormonotherapy with tamoxifen). In 2005, the patient developed leucocytosis associated with asthenia and febrile syndrome and the diagnosis of therapy-related AML was established (AML-M4 according to the French-American-British classification). Blood count was hemoglobin 11.0 g/dL, platelets 102 × 10^9^/L, and leukocytes 71 × 10^9^/L with 13% circulating blasts and the bone marrow was infiltrated with 41.3% blasts. She was treated with chemotherapy (cytarabine, daunorubicin, and cyclosporin) and a complete response was attained. The patient was proposed to bone marrow transplantation, but no compatible donor was found. Ten months later the patient showed evidence of relapse with leukocytosis and thrombocytopenia and died within five months.

**Figure 1:**
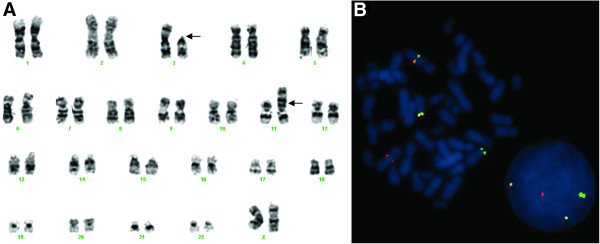
**Cytogenetic and fluorescence in situ hybridization studies of the AML patient.** The bone marrow sample was cultured for 24 h following standard procedures. Chromosome banding analysis of the bone marrow was performed after Leichmann’s staining and the karyotype was described according to ISCN [4]. **A**) Bone marrow karyotype obtained by GTL banding technique exhibiting a t(3;11)(p11;p15) as the sole chromosome abnormality, suggesting the involvement of the *NUP98* gene located at 11p15. Derivative chromosomes 3 and 11 are marked with arrows. BAC clones for *NUP98* and *POU1F1* were selected using the UCSC Human Genome Browser and obtained from the BACPAC Resource Center. **B**) Dual color, dual fusion FISH analysis performed on metaphases using BAC probes for *NUP98* (CTD-3082 N2, RP11-161I4) labeled in green and for *POU1F1* (CTD-2372C6) labeled in red, showed the presence of fusion signals on der(3) and der(11) and isolated red and green signals on normal chromosomes 3 and 11, respectively.

The bone marrow karyotype revealed a t(3;11)(p11;p15) as the sole cytogenetic abnormality in all 30 metaphases analyzed (Figure 1A), suggesting the involvement of the *NUP98* gene located in 11p15. The previous knowledge that HD transcription factors are frequently involved in rearrangements with the *NUP98* gene, combined with a GenBank search of putatively expressed genes on chromosomal band 3p11, prompted us to hypothesize that the *NUP98* fusion partner was *POU1F1*. FISH analysis revealed the presence of fusion signals on the der (3) and on the der (11) chromosomes, strongly suggesting the presence of a rearrangement involving *NUP98* and *POU1F1* (Figure 1B).

**Figure 2:**
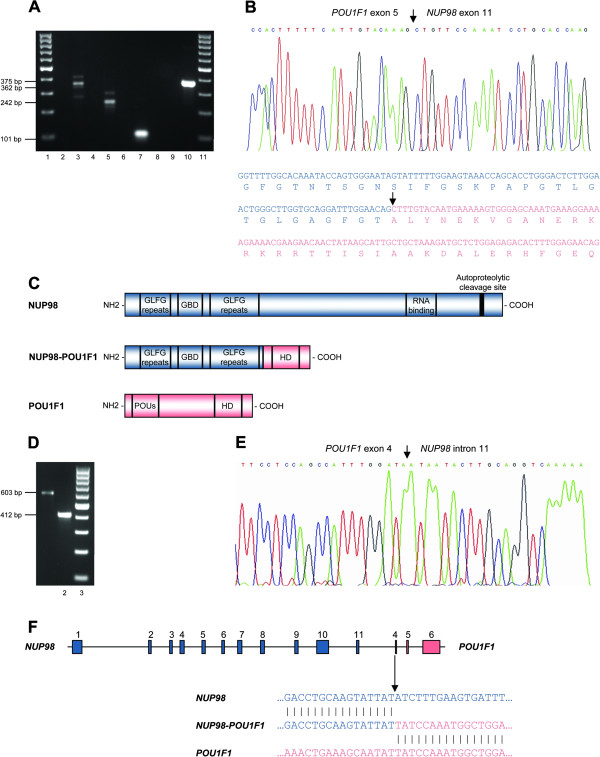
**Characterization of the *NUP98-POU1F1 *fusion. A**) RT-PCR analysis with one antisense primer on *POU1F1* exon 5 and three sense primers on *NUP98* exons 9, 10, and 11 (lanes 3, 5, and 7) showed the presence of PCR fragments of 362, 242, and 101 bp, respectively, suggestive of a fusion between *NUP98* exon 11 and *POU1F1* exon 5. Additional RT-PCR analysis with several *NUP98* and *POU1F1* primer combinations gave additional support to this hypothesis since no amplification was observed (lanes 2, 4, 6, 8, and 9). Lane 10: RNA integrity check (370 bp *B2M* gene fragment). Lanes 1 and 11: 100 bp molecular marker. **B**) Sequence analysis followed by a BLAST search confirmed that *NUP98* exon 11 was fused in-frame with nucleotide 730 of the *POU1F1* transcript (arrow). **C**) Schematic representation of the NUP98, POU1F1, and the NUP98-POU1F1 fusion proteins showing the relevant domains of the partner and chimeric proteins. **D**) Genomic breakpoint analysis with the NUP98_Fint11H sense primer in combination with the POU1F1_Rint4B and POU1F1_Rint4A antisense primers gave origin to amplification products of 603 and 412 bp, respectively (lanes 1 and 2). Lane 3: 100 bp molecular marker. **E**) Sequencing of the amplification products showed that the breakpoint was located 7490 bp downstream of *NUP98* exon 11 and 129 bp downstream of the start of *POU1F1* exon 4 (arrow). **F**) Schematic representation of the genomic DNA breakpoint (arrow) and nucleotide sequence of the genomic breakpoint of the translocation t(3;11) and corresponding normal chromosomes 3 and 11.

RT-PCR with an antisense primer located on *POU1F1* exon 5 and three *NUP98* sense primers located on exons 9, 10, and 11 (Additional file 1) showed the presence of PCR fragments suggestive of an *NUP98-POU1F1* rearrangement resulting from fusion of *NUP98* exon 11 with *POU1F1* exon 5 (Figure 2A). Additional RT-PCR analysis with sense primers located on *NUP98* exons 9, 10, 11 and an antisense primer located on *POU1F1* exon 4 and with a sense primer located on *NUP98* exon 12 and antisense primers located on *POU1F1* exons 4 and 5, gave additional support to this hypothesis, since no amplification was observed (Figure 2A). Sequencing of the amplification products followed by a BLAST search confirmed that *NUP98* exon 11 was fused in-frame with nucleotide 730 of the *POU1F1* transcript (GenBank accession no. NM_000306) (Figure 2B). For the detection of the reciprocal *POU1F1-NUP98* transcript a *POU1F1* sense primer and a *NUP98* antisense primer located on exons 4 and 12, respectively, were used but no fusion transcript could be detected (results not shown). This fusion is expected to give rise to a chimeric fusion protein where the N terminus of NUP98 is fused to the C terminus of POU1F1 (Figure 2C). This is in agreement with the results of all previously reported NUP98 fusions, in which the C-terminal DNA binding homeodomain of the homeobox protein is retained in the fusion protein and the GLFG repeats of NUP98 replace the transactivation domain [1].

For the identification of the genomic breakpoints of the *NUP98-POU1F1* fusion, several primers were designed in *NUP98* intron 11 and *POU1F1* intron 4 (Additional file 1). When the NUP98_Fint11H sense primer was used in combination with the POU1F1_Rint4A and POU1F1_Rint4B antisense primers, amplification products of 412 bp and 603 bp were observed, respectively (Figure 2D). Partial sequencing of the amplification products showed that the breakpoint was located 7490 bp downstream of *NUP98* exon 11 and, interestingly, within *POU1F1* exon 4, 129 bp downstream of the start of *POU1F1* exon 4, and no evidence of mutation or deletion was detected in the breakpoint region (Figure 2A and F). This leads to retention in the genomic sequence of 36 nucleotides from *POU1F1* exon 4 that are not included in the mature *NUP98-POU1F1* messenger RNA, probably as a result of the removal of the splice acceptor site of *POU1F1* intron 3.

Since *FLT3*-ITD mutations have been reported in more than 50% of the patients with *NUP98-*HOX fusions [5], we have searched for this abnormality in our patient using the *FLT3* Mutation Assay for Gel Detection (*InVivo*Scribe Technologies, San Diego, USA) according to the manufacturer’s instructions. We found an internal tandem mutation of the *FLT3* gene, as shown by the presence of an amplification product of approximately 350 bp (Additional file 2: Figure S1).

To our knowledge, this is the first time that *POU1F1*, a POU class 1 homeobox gene, is reported as being involved in a fusion gene in human cancer. *POU1F1* belongs to the POU family of transcription factors that plays a fundamental role in inhibition and promotion of cell differentiation, as well as in the determination of cell lineage and regulation of cell migration, survival and terminal differentiation [6]. In adults, *POU1F1* is expressed in cells of the anterior pituitary gland, where it plays a role in cellular commitment, differentiation and proliferation, driving the expression of growth hormone, prolactin, and thyroid-stimulating hormone β chain genes [6]. *POU1F1* expression has also been reported in hematopoietic and lymphoid tissues [7], and its expression was correlated with increased cellular proliferation in breast cancer and human myeloid leukemic cells, leading to the suggestion that *POUF1* may be involved in the regulation of cellular proliferation [8-10]. Since the expression of *NUP98-*HOX chimeric genes seems to be under the control of the *NUP98* promoter [1], leading to overexpression of the HD transcription factor, it is expected that the same occurs in the rearrangement we here describe, and that *POU1F1* overexpression might result in increased proliferation of leukemic cells.

Several lines of evidence have suggested that many of the NUP98 fusion proteins can act as aberrant transcription factors. It seems that a synergistic action between *NUP98* and the HD partner gene leads to the creation of a unique protein with unique DNA targeting properties and function, which can lead to leukemogenic transformation [1]. Indeed, the *NUP98* chimeric proteins not only retain the N-terminal sequences that are responsible for both DNA binding and transcription activation through a “cryptic” transactivation domain [11-13], but also the C-terminal region of the HD partner can directly bind DNA and activate gene transcription [1]. Furthermore, mouse models of *NUP98-HOX* fusions were shown to induce leukemia with variable latency [1], which was associated with deregulation of HOXA cluster genes that are thought to play a key role in normal hematopoietic differentiation [14]. Impaired terminal differentiation of hematopoietic cells is a hallmark of leukemia and, according to the two-hit model of leukemogenesis, is classified as a type II mutation [15]. However, this model of leukemic transformation, although overly simplified, requires the presence of a concomitant mutation leading to increased proliferation, survival, or both (type I mutation) [15]. It seems that *NUP98-*HOX fusions have the ability to initiate and maintain a state of self-renewal necessary, but not sufficient, for the development of leukemia [1]. Indeed, type I mutations are common in *NUP98* rearranged leukemia, including mutations in the *NRAS, KRAS, KIT, WT1* and *FLT3* genes [5,16]. The type I mutation in the case we here present was the *FLT3*-ITD mutation, which we hypothesize collaborated with the *NUP98-POU1F1* fusion in malignant transformation.

In summary, we have identified *POU1F1* as the *NUP98* fusion partner in therapy-related AML with a t(3;11)(p11;p15). This is the first POU family member identified as a fusion partner in leukemia, and further studies are necessary to uncover the precise role played by this family of genes in this disease.

## Competing interests

The authors declare that they have no competing interests.

## Authors’ contributions

SL and NC designed and performed the research, analyzed the data and drafted the manuscript. SB performed the sequencing and mutation analyses. CC, JV, and LT performed the chromosome banding and molecular cytogenetic studies. JMM clinically assessed the patient. MRT coordinated the study and participated in manuscript writing. All authors read and approved the final manuscript.

## Supplementary Material

Additional file 1 Oligonucleotide primers used in this study. Click here for file

Additional file 2: Figure S1
Detection and analysis of the FLT3-ITD mutation. Lane 1 and 8: 100 bp molecular marker. Lane 2: specimen control size ladder with amplification products of approximately 100, 200, 300, 400 and 600 bp confirming the patient DNA sample integrity. Lanes 3 and 7: no template controls. Lane 4: presence of amplification products of approximately 330 bp (the wild type allele) and a larger amplification product corresponding to the detection of internal tandem duplication (ITD) of the *FLT3* gene. Lane 5: negative control (amplification of polyclonal control DNA of approximately 330 bp). Lane 6: positive control (amplification of clonal control DNA of approximately 360 bp). Click here for file
