# Supplementary material for: POU1F1 is a novel fusion partner of NUP98 in acute myeloid leukemia with t(3;11)(p11;p15)
Source: Mol Cancer. 2013 Jan 18;12:5. doi: 10.1186/1476-4598-12-5 (PMC3567982; doi:10.1186/1476-4598-12-5)
Supplement: Additional file 1 — Oligonucleotide primers used in this study. [file 1476-4598-12-5-S1.doc]

**Additional file 1: Oligonucleotide primers used in this study.**

| **Primer** | **Exon** | **Intron** | **5’-3’ Sequence** | **Ensemble transcript** | **Nucleotide**  **position** |
| --- | --- | --- | --- | --- | --- |
| NUP98_F09 | 9 | - | CAAGCCTCACAGCCTGGAG | ENST00000324932 | 1388_1406 |
| NUP98_F10 | 10 | - | ACCCTGTTTGGCAATAACAAGC | ENST00000324932 | 1508_1529 |
| NUP98_F11 | 11 | - | GGGACTCTTGGAACTGGGCT | ENST00000324932 | 1649_1668 |
| NUP98_F12 | 12 | - | CTTTGGAGCCCCCCAGG | ENST00000324932 | 1804_1820 |
| NUP98_R12 | 12 | - | CTCCAAAGCCCAAAGTGGC | ENST00000324932 | 1793_1811 |
| POU1F1_R4 | 4 | - | TCAGAGCCATGCACAGCTG | ENST00000350375 | 598_616 |
| POU1F1_R5 | 5 | - | CTTATAGTTGTTCTTCGTTTTCTTTTCCTT | ENST00000350375 | 761_790 |
| POU1F1_F04 | 4 | - | GCTGTGCATGGCTCTGAATTC | ENST00000350375 | 600_620 |
| NUP98_Fint11A | - | 11 | CTGCTACCATGAGATCTAGGAAGTGA | - | 9_34 |
| NUP98_Fint11B | - | 11 | GACCCTTCTACCTGCCCAGC | - | 510_529 |
| NUP98_Fint11C | - | 11 | CTTGGCCTCCAAAGTGCTG | - | 1031_1049 |
| NUP98_Fint11D | - | 11 | GTTATGCTTCTAATCCCAGCACTTT | - | 2396_2420 |
| NUP98_Fint11E | - | 11 | TTTAATGAAAATTCCTCATTATACCCCA | - | 4366_4393 |
| NUP98_Fint11F | - | 11 | GGAAATGAGAATTGTGAGTCAGCA | - | 5352_5375 |
| NUP98_Fint11G | - | 11 | TTGTGGACCATTACTTCTTTCATTAAAC | - | 6232_6259 |
| NUP98_Fint11H | - | 11 | CATCCTTACTGATTTATATTGTGTGGGT | - | 7380_7407 |
| POU1F1_Rint4A | - | 11 | AGTATTTTACCCCTTCATGCTGATG | - | 241_265 |
| POU1F1_Rint4B | - | 4 | AGGCTCTGACATTTGTATGAGTAGATAATTT | - | 426_456 |
| B2M_F | 1 | - | ATGTCTCGCTCCGTGGCCTTAGCT | ENST00000558401 | 71_94 |
| B2M_R | 3 | - | CCTCCATGATGCTGCTTACATGTC | ENST00000558401 | 422_445 |
