# Supplementary figures and images for: POU1F1 is a novel fusion partner of NUP98 in acute myeloid leukemia with t(3;11)(p11;p15)
Source: Mol Cancer. 2013 Jan 18;12:5. doi: 10.1186/1476-4598-12-5 (PMC3567982; doi:10.1186/1476-4598-12-5)

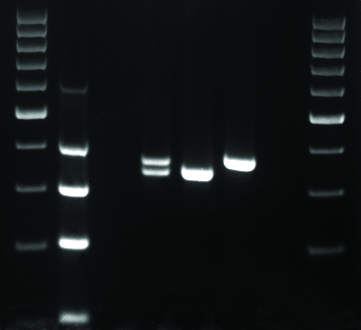

Supplement: Additional file 2: Figure S1 — Detection and analysis of the FLT3-ITD mutation. Lane 1 and 8: 100 bp molecular marker. Lane 2: specimen control size ladder with amplification products of approximately 100, 200, 300, 400 and 600 bp confirming the patient DNA sample integrity. Lanes 3 and 7: no template controls. Lane 4: presence of amplification products of approximately 330 bp (the wild type allele) and a larger amplification product corresponding to the detection of internal tandem duplication (ITD) of the FLT3 gene. Lane 5: negative control (amplification of polyclonal control DNA of approximately 330 bp). Lane 6: positive control (amplification of clonal control DNA of approximately 360 bp). [file 1476-4598-12-5-S2.tiff]
